# Consumer‐orientated development of hybrid beef burger and sausage analogues

**DOI:** 10.1002/fsn3.466

**Published:** 2017-05-03

**Authors:** Michelle Neville, Amparo Tarrega, Louise Hewson, Tim Foster

**Affiliations:** ^1^ Division of Food Sciences Sutton Bonington Campus University of Nottingham Leicestershire LE12 5RD UK

**Keywords:** Acceptability, CATA, consumer studies, hybrid meat analogs, preference mapping

## Abstract

Hybrid meat analogues, whereby a proportion of meat has been partially replaced by more sustainable protein sources, have been proposed to provide a means for more sustainable diets in the future. Consumer testing was conducted to determine consumer acceptability of different formulations of Hybrid beef burgers and pork sausages in comparison with both meat and meat‐free commercial products. Acceptability data were generated using the 9‐point hedonic scale. Check‐all‐that‐apply (CATA) questioning was used to determine the sensory attributes perceived in each product as well as information on the attributes of consumers’ ideal products. It was identified that Hybrid products were generally well liked among consumers and no significant differences in consumer acceptability (p > .05) were identified between Hybrid and full meat products, whereas meat‐free products were found to be less accepted. However, Hybrid sausages received higher acceptability scores (6.00–6.51) than Hybrid burgers (5.84–5.92) suggesting that format may have a large impact on consumer acceptability of Hybrid products. Correspondence Analysis (CA) indicated that Hybrid products were grouped with meat products in their sensory attributes. Penalty analysis found that a “meaty flavor” was the largest factor driving consumer acceptability in both burgers and sausages. Cluster analysis of consumer acceptability data identified key differences in overall acceptability between different consumer groups (consumers who only eat meat products and consumers who eat both meat and meat‐free products). The Hybrid concept was found to bridge the acceptability gap between meat and meat‐free products; however, further product reformulation is required to optimize consumer acceptability.

## 1 INTRODUCTION

Global meat consumption and production has dramatically increased over the years raising growing concerns among governmental bodies, academics, and industry leaders (Cordts, Nitzko, & Spiller, 2014; Graça, Calheiros, & Oliveira, 2015; Speedy, 2003; Tilman, Balzer, Hill, & Befort, 2011). Such a demand is unsustainable and has been identified as the cause of many environmental, health and sustainability related issues (Cordts et al., 2014). The significant challenge of feeding 9 billion people by 2050 poses concerning questions as to how we can meet the predicted demand, sustainably (de Bakker & Dagevos, 2012). It has been suggested by the FAO that we will have to double the production of meat if we are to deliver on the predicted demand for 2050 (Steinfeld et al., 2006). This is alarming, as already competition for agricultural land and resources as well as the unknown impact of climate change on agriculture suggests that we cannot achieve the future protein demand using current practices (de Bakker & Dagevos, 2012). Thus, feeding the future population is a concern that needs addressing sooner rather than later (Godfray et al., 2010; Steinfeld et al., 2006). Besides this, a diet high in animal proteins has been linked to negative health effects of obesity, type 2 diabetes, and an increased risk of heart disease and some types of cancer (Chao et al., 2005; Mann, 2002; Walker, Rhubart‐Berg, McKenzie, Kelling, & Lawrence, 2005).

Growing eastern economies and other developing countries have placed further pressure on the supply and demand of meat with China's demand almost doubling its consumption between 1992 and 2002 (Naylor, 2005). Livestock production is a relatively inefficient process as around 7 kg of grain are required for 1 kg of beef, 4 kg of grain for 1 kg of pork, and 2 kg of grain for 1 kg of poultry (Aiking, de Boer, & Vereijken, 2006). Thus, it is difficult to justify such large use of crops to feed livestock rather than directly to humans.

Converting predominantly meat eaters to a meat‐reduced diet is a societal transition that will require careful strategic planning if we are to shift to more sustainable diets. Although media coverage of the negative side effects of meat consumption have been found to play a major role in reducing meat intake (Burton & Young, 1996; Cordts et al., 2014; Rickertsen, Kristofersson, & Lothe, 2003), only minimal success has been achieved through other nongovernmental organizational campaigns (Laestadius, Neff, Barry, & Frattaroli, 2013). One of the pathways of transition is to achieve partial substitution in the diet of animal proteins with more sustainable proteins such as plant protein (Schösler, de Boer, & Boersema, 2012), although achieving long‐term transitions rather than phases in consumption behavior are key to success (Hoek et al., 2013). The requirement for meat substitution is a topic that has been largely discussed (de Bakker & Dagevos, 2012; Lea, Crawford, & Worsley, 2006), however few studies have quantified what is required by consumers in order for them to change their behaviors (Elzerman, Hoek, van Boekel, & Luning, 2011; Hoek et al., 2011; Schösler et al., 2012). It has been found that to create an effective dietary change, new practices must be somewhat similar to the previous behavior of the consumer (Ryan & Deci, 2000). Convenience and minimal skill in cooking techniques have also been identified as a major factor in hindering consumer transition to alternative protein sources (Schösler et al., 2012). The proposed method in this study to achieve meat substitution is by a built‐in meat reduction in products by partially replacing animal proteins with more sustainable protein sources. Such a strategy would bridge the gap between meat and meat‐free products, provide convenience, and allow consumers to continue using products as they conventionally would.

Meat is an expensive commodity and supermarkets offer a wealth of low‐cost products with low percentages of meat whereby replacement has been achieved through cheap fillers and bulking agents as a means to cut costs. Such fillers have little nutritional bonus and usually consist of cereals, starches, and breadcrumbs (Gunter & Peter, 2007). In this study, a proportion of meat has been replaced with ingredients that contain a high amount of protein as a means to a more sustainable way to include alternative proteins within the diets of consumers.

The meal context in which meat substitutes are used has been found to have a significant impact on consumer acceptability (Elzerman et al., 2011; Schösler et al., 2012). Schösler et al. (2012) assessed current consumer behaviors regarding meat substitution and identified that meal formats played a key role in finding pathways to transition. By combining a meat substitute with a food format familiar to the consumer (mince or pieces), it was proposed that meat substitution in convenience foods, whereby meat as an ingredient is already less visible, posed a suitable method for substitution. Hoek et al. (2013) identified that repeated exposure to meat substitutes increased consumer acceptability. It was suggested that focus should be made on increasing willingness to try meat substitutes and creating positive initial product experiences.

It has previously been identified that nonvegetarian consumers generally judge the overall sensory quality of meat substitutes lower than that of meat (Hoek et al., 2013). The special status of meat within society, and its taste and texture are highly valued by many consumers, especially the juiciness and tenderness (Elzerman et al., 2011). Current meat substitutes are likely to be perceived as less complex than meat as they do not possess the sensory attributes in order to be accepted by meat eaters. Taste and texture have been identified as important characteristics for the acceptance of meat substitutes (Hoek et al., 2013). Although it has been identified that consumers prefer a meat‐like meat substitute (Hoek et al., 2011), mimicking meat—a highly complex product—is a large technological challenge. Thus, in order to create successful meat alternatives, a consumer‐orientated approach to product development is required. One way to achieve this is through developing products with consumer preferences in mind (Grunert and Valli, 2001; Stewart‐Knox and Mitchell, 2003).

Check‐all‐that‐apply (CATA) questions offer an alternative to conventional Quantitative Descriptive Analysis (QDA) methods which are comparatively more expensive and time consuming due to the requirement of trained panels (Meilgaard et al., 1999). CATA questioning has been described as a reliable, quick, and cost effective method of consumer testing and has been gaining popularity for sensory characterization of food products over recent years (Ares, Barreiro, Deliza, Giménez, & Gámbaro, 2010; Ares, Dauber, Fernández, Giménez, & Varela, 2014; Bruzzone et al., 2015; Da Conceição Jorge et al., 2015; Dooley, Lee, & Meullenet, 2010). In this method, consumers are presented with a list of sensory terms and are asked to select all the terms they consider appropriate to describe a sample (Ares et al., 2014). Da Conceição Jorge et al. (2015) used the application of CATA questions to evaluate and characterize samples of “Mortadella,” an Italian pork sausage eaten cold. Ares et al. (2014) used Penalty Analysis on samples of yogurts and apples to link consumer acceptance with a product's sensory characteristics; thereby, identifying the terms that positively or negatively contributed to a products acceptance.

In this study, two meat products (pork sausages and beef burgers, two meal formats familiar to UK meat consumers) with partial meat substitution were tested against commercial meat and meat‐free products in order to determine consumer acceptance in relation to the two categories. Products in which part of the meat is replaced by more sustainable protein sources is not a novel concept and have been termed Hybrid meat analogues. Hybrid sausages, hamburgers, and mince have already entered the Dutch food markets and have created a means whereby eating sustainable products gradually becomes more accessible (de Bakker & Dagevos, 2012). Caparros Megido et al. (2016) assessed the sensory liking of Hybrid insect‐beef burgers. Their studies found that overall liking varied between genders as Hybrid products were preferred by men more than women. Food neophobia (reluctance to try novel foods) was a large contributor to acceptance. However, to the best of our knowledge, no studies have been conducted that assess the sensory attributes and consumer acceptance of hybrid products by meat eaters. In this study, a consumer‐generated lexicon of the sensory terms was produced. Consumers indicated their liking of each product and CATA questioning was used to determine the sensory attributes that characterize the products. Consumers were also asked to indicate the sensory attributes that characterize their ideal pork sausage or beef burger. The combined analysis of liking and CATA allows the identification of drivers for liking and consumer acceptance. Penalty analysis enabled an indication of the penalty on liking when undesirable attributes are present or the sample is different from the ideal. Assessed together with the ideal, directions to aid in product reformulation are outlined.

## 2 MATERIALS AND METHODS

A variety of alternative proteins (textured soya, mycoprotein, insect protein, and pulses) were assessed for hybrid formulations before two were selected. Two concept formulations of Hybrid beef burgers and two formulations of Hybrid pork sausages were produced at pilot scale (DuPont, Denmark), frozen and transported to the United Kingdom and stored frozen (−18 °C ± 2°C). Commercial meat and meat substitutes (Table 1) were purchased from a local supermarket. All samples for consumer testing were prepared on the day of testing, served within 30 min of cooking, and kept warm in slow cookers (75°C ± 4°C). All commercial samples were prepared as per the manufacturer's guidelines. Diluted lime cordial (1:5 lime to water, Rose's Lime Juice Cordial) and mineral water (Evian) were used for palate cleansing before and between samples.

**Table 1:** Concept and commercial samples

Burgers	Products	Cooking method
Concept Formulation	Hybrid 1%–37% Beef Hybrid 2%–37% Beef	Oven cooked Oven cooked
Commercial Products	Beef burger–77% Beef Vegetarian burger 1–Mycoprotein based Vegetarian burger 2–Soya based	Oven cooked Pan fried Oven cooked
Sausages		
Concept Formulation	Hybrid 1%–30% Pork Hybrid 2%–30% Pork	Pan fried Pan fried
Commercial Products	Meat sausages–61% Pork Vegetarian sausage 1–Mycoprotein based Vegetarian sausage 2–Soya based	Pan fried Pan fried Pan fried

### 2.1 Sensory evaluation

All sensory analysis was performed after approval by The University of Nottingham Faculty of Medicine and Health Science Research ethics committee. A consumer‐generated lexicon of sensory attributes for the CATA questions was first defined. Consumers (*n* = 12; M = 5, F = 7), aged 18–60 years who consume both meat and meat substitutes, were recruited from the campus population via email advertisement to attend a 1 hr session. In sensory booths, each consumer received three pairs of sausage samples and were asked to write down differences in sensory attributes relating to texture, flavor, and appearance. After a 5‐min rest break, consumers were presented with three pairs of burger samples and asked to do the same. Sample pairs were selected to represent the extremes in differences in sensory attributes as well as to illustrate all attributes within the sample set of Meat, Hybrid, and Vegetarian products. Frequency tallies were performed and the most recorded terms (Table 2) were used to develop the CATA questionnaire.

**Table 2:** Consumer‐generated sensory attributes relating to texture, flavor, and appearance describing the sample set

	Burger products	Sausage products
Texture	Juicy	Dry
	Dry	Fibrous
	Granular	Soft
	Greasy	Hard
	Easy to cut	Easy to cut
	Difficult to cut	Difficult to cut
	Hard	Greasy
	Soft	Poor mouthfeel
		Moist
Flavor	Sweet	Meaty
	Peppery	Wheaty
	Smokey/Grill	Herby
	Off‐flavor	Peppery
	Meaty	Off‐flavor/Unpleasant aftertaste
	Wheaty	
Appearance	Dark brown color	Dry
	Light brown color	Coarse
	Dry	Visible herbs
	Oily	Pale color
	Processed	Fatty
	Uneven color	

In a second stage, consumers (*n* = 94; M = 43, F = 51) were recruited from the campus population via email and poster advertisements to attend one 30‐min session. Consumers were selected based on their meat consumption behavior and divided into two groups: only meat eaters and do not consume meat substitutes (*n* = 49); most commonly eat meat products but sometimes eat meat substitutes (*n* = 45), and their interest and availability to participate. Consumers received each of the five burger samples and each of the five sausage samples and were asked to consume no more than a quarter of each sample. Samples were presented monadically, on white paper plates labeled with random three digit codes and served at 75°C (±5°C). The order of presentation of samples and tests followed a randomized balanced design.

For each sample, consumers were first asked to score their overall liking using a vertical 9‐point hedonic scale anchored at “dislike extremely” (1) and “like extremely” (9). Next, they completed a CATA questionnaire with the 20 terms related to the sensory attributes of the samples (Table 2). Consumers were asked to try the sample and then check all the terms they considered appropriate to describe each sample. Consumers were also asked to complete the CATA questionnaire to describe their ideal pork sausage and beef burger.

All testing was performed in separate, purpose‐built sensory testing booths, under Northern Hemisphere lighting and under controlled air, temperature, and humidity conditions.

### 2.2 Data analysis

Overall acceptability scores were analyzed using ANOVA one‐factor analysis of variance. Tukey's honestly significantly different (HSD) post hoc analysis of the difference categories with a confidence of 95% was used to identify significant groups in acceptability between samples. Agglomerative Hierarchical Cluster (AHC) analysis was performed in order to identify consumer groups with different preference patterns.

Frequency of use of each sensory attribute in the CATA questionnaire was determined by counting the number of consumers that used that term to describe each sample. Cochran's *Q* test was carried out to identify the significant differences between samples for each of the terms included in the CATA questionnaire.

Correspondence analysis (CA) was used to generate a biplot representing the samples and the relationship between samples and the terms from the CATA questioning.

Penalty analysis was carried out on consumer responses to determine the drop in overall acceptability associated with deviation from the ideal for each of the sensory attributes in the CATA question.

Multiple factor analysis was used to investigate the relationship between responses to the CATA questions and the consumer groups identified in the cluster analysis.

A significance level of 0.05 was chosen and statistical analysis was performed using XLStat–Pro (Addinsoft, France).

## 3 RESULTS

### 3.1 Consumer evaluation of beef burger products

#### 3.1.1 Overall liking

Significant differences in acceptability between beef burger samples were identified (*F *=* *53.636, *p *<* *.0001). As shown in Table 3, acceptability scores of vegetarian and meat‐containing products were varied among consumers. The Vegetarian burger 2 had the lowest acceptability score of 2.85 and was disliked very much by meat‐eating consumers. Tukey's test identified this as significantly different from the other samples. This was followed by Vegetarian burger 1 which received the second lowest acceptability score (5.38), then the Hybrid 2 and Hybrid 1 with mean acceptability scores of 5.84 and 5.92, respectively. Receiving the highest acceptability score of 6.34, corresponding to ‘liked slightly’ was the Meat burger. According to Tukey's test, no significant difference in acceptability was identified between the full meat burger and the two Hybrids; however, a significant difference in acceptability was identified between the meat‐free samples and the meat only sample.

**Table 3:** Mean acceptability scores of burger samples evaluated

Sample	Mean acceptability score
Vegetarian burger 2	2.85^a^ ± 1.60
Vegetarian burger 1	5.38^b^ ± 2.29
Hybrid 2	5.84^b,c^ ± 1.80
Hybrid 1	5.92^b,c^ ± 1.79
Meat burger	6.34^c^ ± 1.66

Mean acceptability scores with different superscripts are significantly different according to Tukey's HSD test with a confidence level of 95%.

#### 3.1.2 CATA questionnaire

##### 3.1.2.1 CATA counts

The frequencies by which consumers checked an attribute for a particular sample are shown in Table 4. As can be seen, samples vary largely in their sensory attributes and significant differences in 19 out of the 20 attributes were identified between samples (*p *<* *.05). No significant difference in “peppery flavor” was identified between the five samples tested (*p *>* *.05). The Vegetarian burger 2 was described as having an “off‐flavor”, “processed appearance,” “wheaty flavor,” “hard texture,” “dry texture,” and being “difficult to cut.” The Vegetarian burger 1 was described as being “juicy,” “easy to cut,” “soft,” having a “processed appearance,” and a “smokey‐grill flavor.” The Hybrid 2 burger was described as “granular” in texture, “easy to cut,” “dark brown” in color, and “meaty” in flavor. The Hybrid 1 and Meat burger were found to be similar in the sensory attributes and were described as “meaty” in flavor, “easy to cut” but having a “dry appearance”. The ideal burger was described as “juicy,” “easy to cut,” and “dark brown” in color with a “meaty flavor.”

**Table 4:** Frequency by which consumers used the terms of the CATA question to describe the burger samples tested and their ideal products. Cochran's Q test identifies significant differences between samples

Attribute	Sample						
	*p*‐value	Vegetarian burger 2	Vegetarian burger 1	Hybrid 2	Hybrid 1	Meat burger	Ideal
Juicy	<.001	1	57	31	25	26	82
Dry Texture	<.001	66	15	32	53	50	1
Granular	<.001	41	6	44	37	38	11
Greasy	−.001	9	27	23	12	11	15
Easy to cut	<.001	31	87	50	68	57	78
Difficult to cut	<.001	35	2	26	12	18	3
Hard	<.001	43	0	24	19	21	6
Soft	<.001	17	85	41	46	41	67
Dark brown color	<.001	50	53	68	50	16	63
Light brown color	<.001	28	24	9	29	58	18
Dry appearance	<.001	50	33	41	59	61	12
Oily appearance	<.001	21	16	30	6	6	25
Processed appearance	<.001	58	50	28	31	37	7
Uneven color	−.001	27	26	10	16	31	2
Sweet	−.004	13	27	15	11	21	16
Peppery	−.072	24	10	19	18	22	47
Smokey Flavor/Grill	<.001	40	81	15	18	17	51
Off‐flavor	<.001	56	18	12	11	6	0
Meaty flavor	<.001	3	40	66	59	69	91
Wheaty flavor	<.001	38	18	19	20	16	3

**Figure 1:**
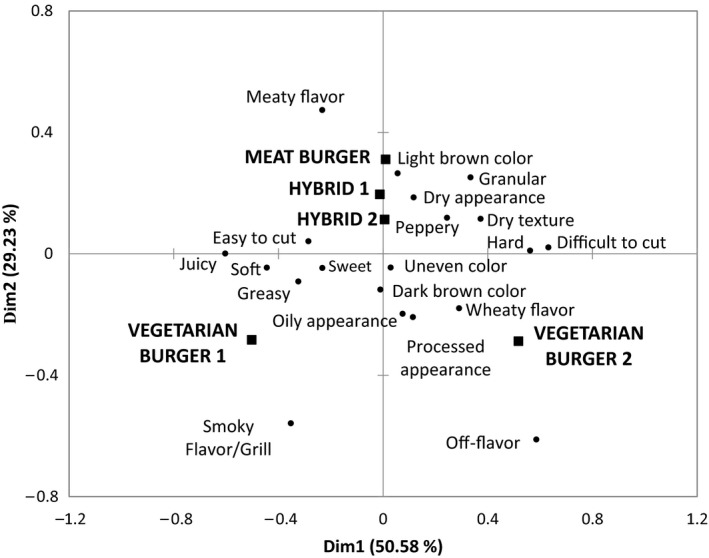
Representation of burger samples and their related terms from the CATA question. First and second dimensions of the correspondence analysis

Correspondence Analysis (CA) is a statistical technique that can be used to generate a biplot showing the relationships between samples and the terms used in CATA questioning. The outcomes of the correspondence analysis of CATA data are shown in Figure 1. The five burger samples were sorted into three areas according to their sensory attributes. The first area comprised the meat‐containing samples; the Meat burger and the two Hybrid products are separated from the nonmeat products along dimension 2. The two Hybrid samples also shared similar formulations and the only contributing factor to differing sensory attributes would have come from the meat replacer used. The Vegetarian burger 1 and the Vegetarian burger 2 have very different formulations; thus, large differences in sensory attributes were identified among consumers and were separated along Dim1. These two samples were separated by the Vegetarian burger 2 product having a “wheaty flavor” and the Vegetarian burger 1 being softer and “juicy,” and having a “smokey flavor.”

##### 3.1.2.2 Penalty analysis

**Figure 2:**
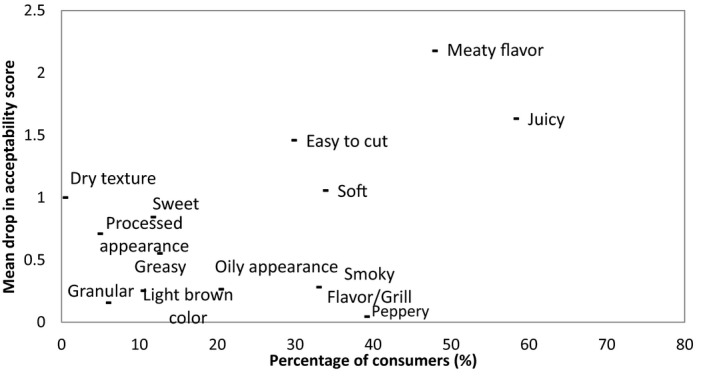
Mean drops in overall acceptability when a sensory attribute was described in a consumer's ideal but when not present in a particular sample, consumer acceptability significantly decreased

Penalty analysis (PA) is a method of determining the penalty or reward on liking scores associated with the presence or intensity of sensory attributes. It is commonly used with liking scores and data from Just‐About‐Right or intensity scales; however, recent studies have utilized this approach with the binary responses (checked or unchecked) from CATA questions (Ares et al., 2014; Plaehn, 2013). PA can also be used to identify directions for product improvements in terms of reformulation if a consumer's “ideal” product is included in the questionnaire (Ares et al., 2014). PA determines the mean drop in consumer acceptability when consumers select an attribute for the ideal products but is not described for the test sample. This data can be used to prioritize product development areas to those which are subject to the highest penalty if not deemed by the consumer to be correct. The results of the penalty analysis to determine the sensory attributes that drive consumer acceptability in burgers are shown in Figure 2. As can be seen, the absence of a “meaty flavor” is found to be the largest contributor to a decrease in consumer acceptability with a drop of 2.20 in acceptability and is related to 47% of consumer responses. The Meat burger received the most counts for “meaty flavor” (Table 4) which would offer an explanation as to why this burger achieved the highest acceptability score. Also, “juicy,” “easy to cut,” and “soft”— all had a large influence in consumer acceptability with mean drops of 1.65, 1.50, and 1.05, respectively. The Vegetarian burger 1 received the most counts for “juicy,” “easy to cut,” and “soft”, thus, increasing its acceptability among consumers. However, the low counts for “meaty flavor” may have prevented the Vegetarian burger 1 from achieving a higher acceptability score. As shown in Table 4, the two hybrid samples and the Meat burger received similar counts for “juicy,” “easy to cut,” and “soft” but the Meat burger received a higher count for “meaty flavor.” Therefore, in order to improve consumer acceptability of the hybrid concepts, reformulation may involve the development of a meatier flavor closer to a consumer's ideal count.

**Figure 3:**
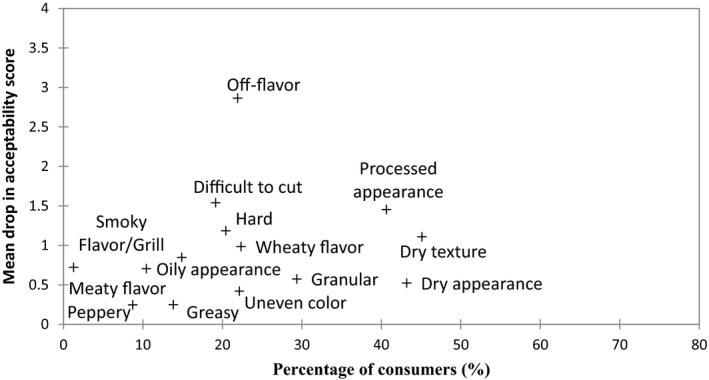
Mean drop in overall acceptability when a sensory attribute was not described in a consumer's ideal and when present in a sample

Figure 3 details the sensory attributes that a sample must not have; otherwise, consumer acceptability significantly decreases. These are the sensory attributes that consumers did not mention in their ideal but when present in a sample, acceptability significantly decreased. “Off‐flavor” “processed appearance,” and “dry texture” were identified as resulting in the largest mean drop in acceptability score with drops of 2.90, 1.40, and 1.10, respectively. The Vegetarian burger 2 received the most counts for all three of these attributes and would provide an explanation as to why this product received such a low consumer acceptability score.

##### 3.1.2.3 Multiple factor analysis

**Figure 4:**
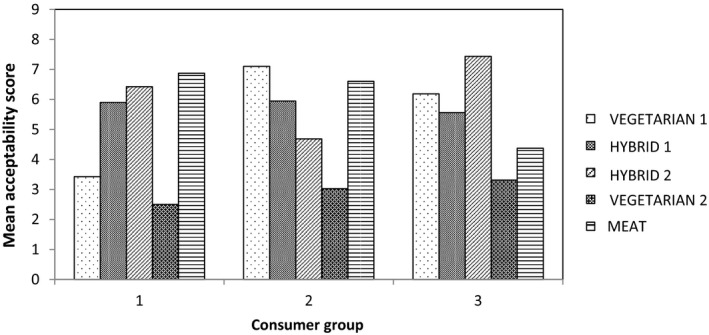
Preferences of the consumer groups identified from the cluster analysis

Cluster analysis was used to identify trends in consumer responses and three significant groups in terms of consumer preferences were identified, their acceptability profiles for the product set are shown in Figure 4. Consumer group 1 (*n* = 40) had a higher preference for the meat‐containing samples, especially the Meat burger, and rejected both Vegetarian burgers. Consumer group 2 (*n* = 38) had a higher preference for both meat and meat‐free products, especially Vegetarian burger 1 and the Meat burger. Consumer group 3 (*n* = 16) were found to have a preference for the two Hybrid samples and Vegetarian burger 1. By identifying individual panelist numbers within each consumer group, group 1 was identified as predominantly the consumers who only eat meat products and do not eat meat substitutes. Group 2 was identified as consumers who most commonly eat meat but sometimes eat meat substitutes. Group 3 was identified as a mixture of the two.

**Figure 5:**
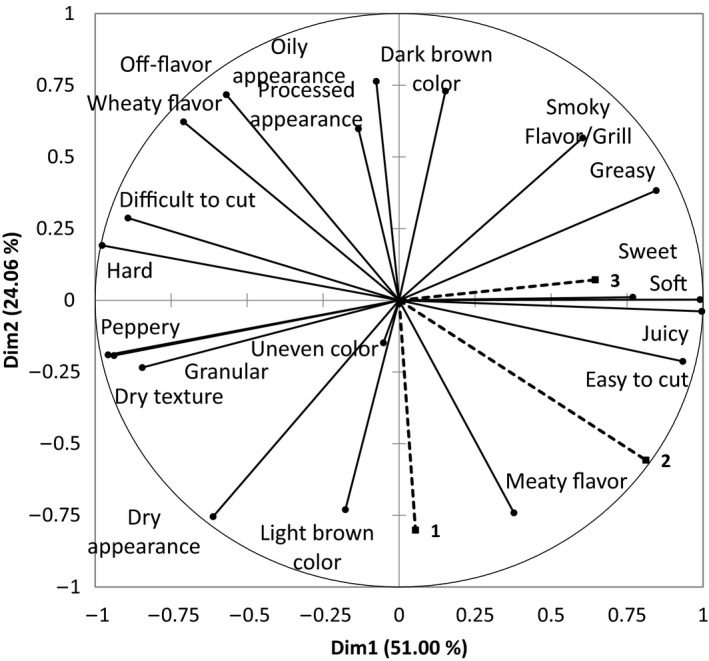
Multiple factor analysis of sensory attributes from CATA questioning and the consumer groups identified from cluster analysis

Multiple factor analysis was used to investigate the relationship between responses to the CATA questions of the consumer groups identified in the cluster analysis (Figure 5). This suggests that the preferred attributes for consumer group 1 (the meat eaters) include “light brown color” and “meaty flavor”, whereas consumer group 2 have a higher preference for the attributes “easy to cut,” “juicy,” and “soft”.

### 3.2 Consumer evaluation of pork sausage products

#### 3.2.1 Overall liking

Significant differences in acceptability scores between pork sausage products were identified (*F *=* *53.636, *p < *.0001). As shown in Table 5, acceptability of meat‐free and meat‐containing products were varied among meat‐eating consumers. The Vegetarian sausage 2 received the lowest mean acceptability score of 4.39. The Vegetarian sausage 1 received the second lowest mean acceptability score of 5.10. According to Tukey's test, these two meat‐free products were identified as significantly different in acceptability from the meat‐containing samples. The Hybrid 2 sausage received a lower acceptability score than the Meat sausage of 6.00 and 6.39, respectively. The Hybrid 1 sausage received the highest mean acceptability score of 6.51 and was ‘liked slightly’ by consumers. However, according to Tukey's test, was not identified as significantly different in acceptability to the Meat and Hybrid 2 sausage.

**Table 5:** Mean acceptability scores of sausage samples evaluated

Sample	Mean acceptability score
Vegetarian sausage 2	4.39^a ^± 2.07
Vegetarian sausage 1	5.10^a ^± 1.97
Hybrid 2	6.00^b ^± 1.52
Meat sausage	6.39^b ^± 1.78
Hybrid 1	6.51^b ^± 1.52

Mean acceptability scores with different superscripts are significantly different according to Tukey's test with a confidence level of 95%.

#### 3.2.2 CATA questioning

##### 3.2.2.1 CATA counts

The frequencies by which consumers checked a sensory attribute for each of the sausage products including their ideal are shown in Table 6. Samples were all described very differently in their sensory attributes and 18 out of the 20 attributes were identified as being significantly different between samples (*p *<* *.05). “Peppery flavor” and “wheaty flavor” were identified as not significantly different (*p *>* *.05) between the five products. However, similarities were identified between the two Hybrid sausages. The Hybrid products were identified as having the “meatiest color” and a “meaty flavor” in line with the Meat sausage which received the highest counts for “meaty flavor”. The two Hybrids were also described as having the “driest texture”. These similarities would be expected as the recipes used for the two Hybrids are the same with the only differing factor being the meat replacer used. The Hybrid 1 sausage was described as “easy to cut,” having a “fatty appearance,” and received low counts for “off‐flavors”. The Hybrid 2 sausage was described as being “difficult to cut,” “hard,” received low counts for “greasiness,” “off‐flavor,” and had a “pale color.” The Vegetarian sausage 2 was described as having a “fibrous texture”, “being easy to cut”, “herby” in flavor and received the highest counts for an “unpleasant aftertaste and off‐flavor”; however, in general, these counts were quite low. The Vegetarian sausage 1 was described as “easy to cut,” having a “moist texture,” and very “soft”. The Meat sausage was described as “greasy,” “fatty appearance,” “easy to cut,” “moist,” having a “coarse texture” and “pale in color”. The ideal sausage was described as “easy to cut”, having a “moist texture”, a “meaty color” and “meaty flavor”.

**Table 6:** Frequency by which consumers used the terms of the CATA question to describe the sausage products tested and their ideal products. Cochran's *Q* test identifies significant differences between samples

Attribute	Sample						
	*p*‐value	Vegetarian sausage 2	Vegetarian sausage 1	Hybrid 2	Meat sausage	Hybrid 1	Ideal
Fibrous texture	<.001	49	17	35	5	23	15
Dry texture	<.001	23	6	32	0	23	2
Poor mouthfeel	−.003	35	26	26	16	15	0
Greasy	<.001	6	20	14	62	14	16
Easy to cut	<.001	53	75	27	66	53	72
Difficult to cut	<.001	11	1	49	10	20	1
Hard	<.001	9	0	36	2	15	7
Soft	<.001	45	77	29	74	46	60
Moist texture	<.001	28	59	19	73	33	75
Coarse appearance	<.001	23	11	21	2	13	18
Dry appearance	<.001	32	29	30	0	27	9
Visible herbs	<.001	76	20	27	0	16	42
Pale color	<.001	34	38	18	47	19	2
Meaty color	<.001	18	21	51	39	57	78
Fatty appearance	<.001	3	13	18	52	14	15
Herby flavor	<.001	65	39	35	15	28	40
Peppery flavor	−.255	35	43	31	31	35	38
Off‐flavor/unpleasant aftertaste	<.001	32	29	10	4	6	0
Meaty flavor	<.001	13	21	50	59	55	82
Wheaty flavor	−.109	23	29	24	14	23	2

**Figure 6:**
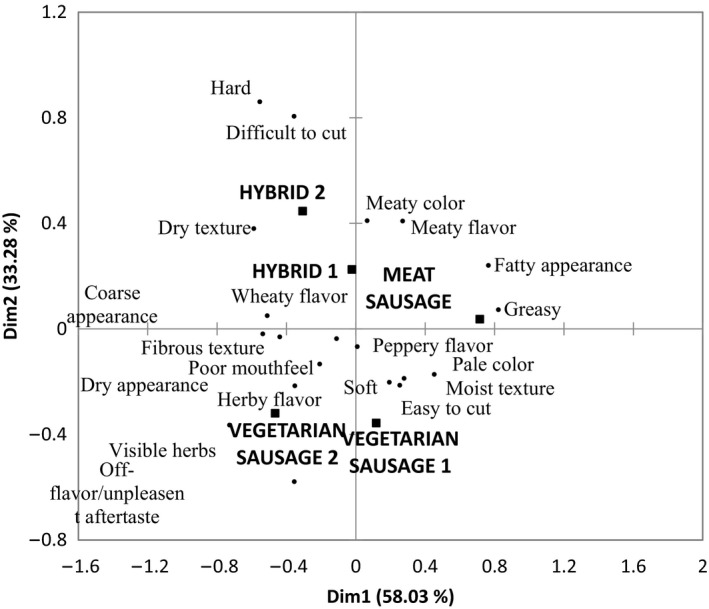
Representation of sausage products and their related terms from the CATA question. First and second dimensions of the correspondence analysis

The outcomes of the correspondence analysis of CATA data generated are reported in Figure 6. The correspondence analysis shows that samples were found to be very different in their sensory attributes; however, similarities between the two Hybrids were identified and thus were grouped together due to their similar formulations. The two Vegetarian sausages are shown to be very different in their sensory attributes which is due to their very different formulations and are separated along dimension 1.

##### 3.2.2.2 Penalty analysis

**Figure 7:**
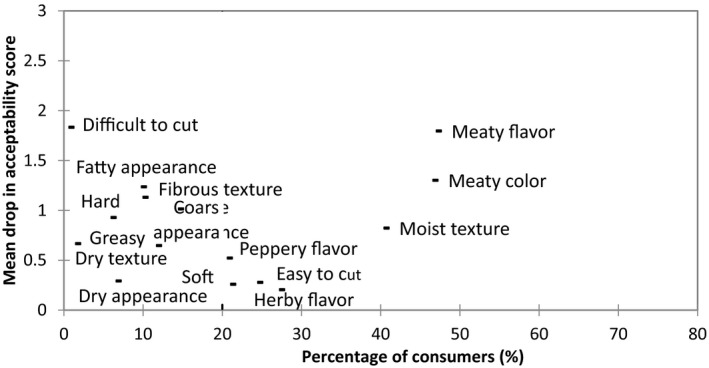
Mean drop in overall acceptability when a sensory attribute was described in a consumer's ideal but when not present in a particular sample, consumer acceptability significantly decreased

The results of the penalty analysis of the attributes that help to drive consumer acceptability in pork sausages are shown in Figure 7. It identified that “meaty flavor,” “meaty color,” and “moist texture” were important factors for consumer acceptability and account for a drop in overall consumer acceptability of 1.80, 1.30, and 0.80, respectively, with 47%, 46% and 40% of consumers agreeing with this trend, respectively. The Meat sausage was described as “meaty” in flavor with a “moist texture” but was described as “pale”, whereas the Hybrid 1 sausage was described as having a “meaty flavor” and color, thus, showing the importance of a products color in influencing consumer acceptance.

**Figure 8:**
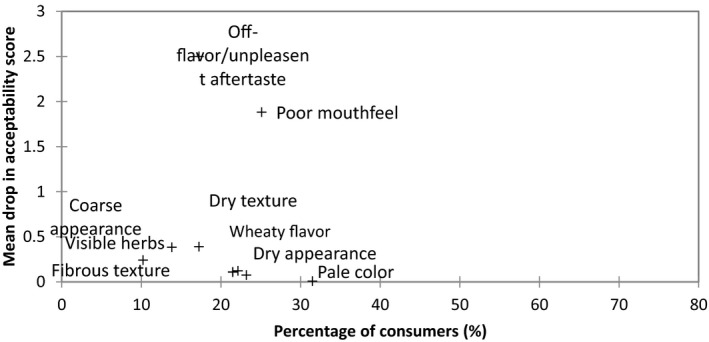
Mean drop in overall acceptability when a sensory attribute was not described in a consumer's ideal and when present in a sample

Figure 8 reports on the sensory attributes that a sample must not have; otherwise, consumer acceptability significantly decreases. “Off‐flavor/unpleasant aftertaste” and “poor mouthfeel” were identified as the most important sensory attributes resulting in a large drop in overall acceptability with scores of 2.50 and 1.90, respectively. The Vegetarian sausage 2 received the most counts for “off‐flavor/unpleasant aftertaste” and “poor mouthfeel” which would explain its low acceptability score.

##### 3.2.2.3 Multiple factor analysis

**Figure 9:**
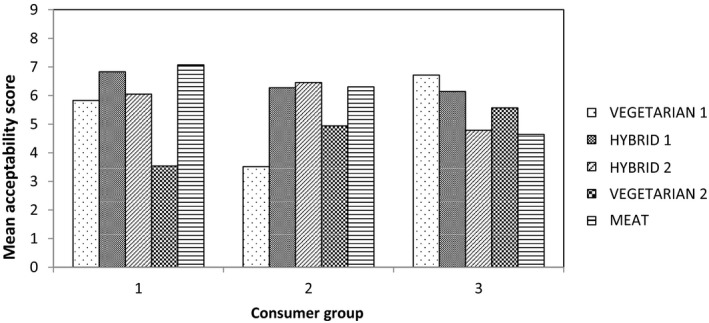
Preferences of the consumer groups identified from the cluster analysis

Cluster analysis identified three significant groups in consumer behaviors in terms of preference (Figure 9). Consumer group 1 (*n* = 41) were identified as having a preference for both meat and meat‐free products with the exception of the Vegetarian sausage 2. This group, however, had a higher preference for the Meat and Hybrid 1 sausage but Hybrid 2 and Vegetarian sausage 1 received similar acceptability scores. Consumer group 2 (*n* = 33) were found to have a higher preference for the meat‐containing samples; the two Hybrids and the Meat sausage, due to the “meaty color” and “meaty flavor”. Consumer group 3 (*n* = 14) were found to have a higher preference for the Vegetarian 2 and Hybrid 1 sausages.

By identifying individual panelist numbers within each group, group 1 were identified as predominantly the consumers who tend to like both meat and meat‐free products. Group 2 were identified as predominantly pure meat eaters; they are the consumers who only eat meat products and do not consume alternatives or substitutes. Group 3 were identified as a mix of the two.

**Figure 10:**
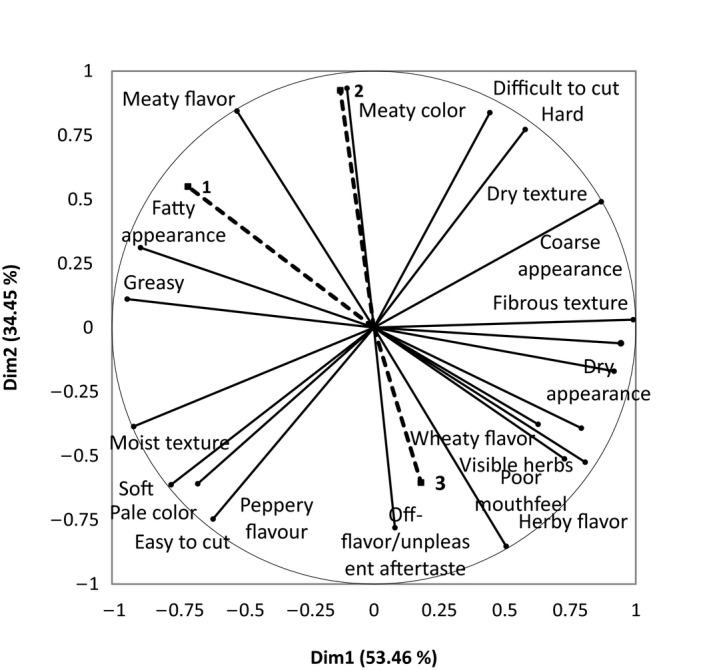
Multiple factor analysis of sensory attributes from CATA questioning and the consumer groups identified from cluster analysis

Multiple factor analysis (Figure 10) identified the relationship between responses to the CATA questions of consumer groups identified during the cluster analysis. This suggests that consumer group 1 has a higher preference for samples containing the sensory attributes “greasy,” “fatty appearance,” and “meaty flavor”, whereas consumer group 2, the meat eaters, has a higher preference for products that have a “meaty flavor” and “meaty color.”

## 4 DISCUSSION

The importance of meat alternatives has been well documented (de Bakker & Dagevos, 2012; Lea et al., 2006). Modern day demand and consumption of meat is unsustainable and a need to reduce meat consumption has importance for both the environment and human health. Although novel protein alternatives are widely available on the market, the lack of acceptability of some meat substitutes with meat‐eating consumers due to a perceived compromise in sensory attributes, has hindered consumer transitions to more sustainable diets (de Bakker & Dagevos, 2012). A means to create a stepping stone between meat and meat‐free is through Hybrid meat analogues, creating products with greater consumer acceptability but reduced meat content. This should aid in lowering the impact on both human health and the environment.

In this study, a consumer‐generated lexicon of the sensory attributes that compromise the products was generated for two sets of products; beef burgers and pork sausages. This sensory lexicon in a consumer's language was used in Check‐all‐that‐apply (CATA) analysis. Consumers were presented with samples of commercial meat, meat‐free, and Hybrid products and scored overall liking. Using the CATA questionnaire, they identified the sensory attributes they perceived to be present in each product as well as indicating the attributes of their ideal product. The results found that Hybrid products are generally well liked among consumers. However, it was found that Hybrid sausages had a higher overall acceptability in comparison with Hybrid burgers suggesting that the format of the product may have a large impact on consumer acceptability.

No significant differences in consumer acceptability (*p *>* *.05) could be identified between meat and Hybrid products, whereas consumer acceptability of meat‐free products was significantly lower than the meat‐containing products (*p *<* *.05).

Correspondence analysis showed that the Hybrids were grouped together with the full meat products indicating that they possess similar sensory attributes. By clustering acceptability data it was also identified that significant differences in acceptability of the products tested existed between different consumer groups. Predominantly meat eaters who do not eat meat substitutes have a higher preference for the meat‐containing products. Consumers who most commonly eat meat but also eat meat substitutes were found to have a broader preference for both meat‐containing and meat‐free products suggesting that familiarity to vegetarian meat substitutes increased their acceptability among this consumer group. As has been previously suggested (Hoek et al., 2011, 2013), in this study, multiple factor analysis suggests that replicating a “meaty flavor” and “meaty color” in Hybrid products is key to increasing their acceptability among predominantly meat consumers. However, in terms of converting the three consumer groups to a meat‐reduced diet, it is encouraging to see that at least one of the Hybrid formulations is prominent within each group. Thus, it could be proposed that by creating a positive initial experience and replicating the flavor and texture of meat within a substitute and repeated exposure of Hybrid products among meat consumers will aid in the transition to more sustainable diets.

The novel approach used in this study of combing Penalty analysis with CATA data helped to uncover, in a consumer language, the key attributes that drive consumer liking and disliking in meat‐containing and meat‐free products. This can provide information and focus for product reformulation. CATA questioning is a relatively novel consumer analysis technique and offers an alternative to conventional Quantitative Descriptive Analysis (QDA) (Meilgaard, Carr, & Civille, 2006). CATA questioning provides a rapid and easy method of sensory analysis using consumer language. Using appropriate analysis techniques, a wealth of information can be generated to help drive product reformulation. However, a disadvantage of CATA questioning is related to the fact that information about an attributes intensity and degree of difference between a product and the ideal cannot be generated (Ares et al., 2014).

The results generated from this study indicate that the Hybrid concept helps to bridge the acceptability gap among predominantly meat eaters between meat and meat‐free products. It is possible that the Hybrid concept could be used as a stepping stone in the transition of converting meat eaters to a meat‐reduced diet, increasing their familiarity with meat substitution. The Hybrid concept does not provide the sole means to solving the protein issue but should be used among various other strategies to move consumers to more sustainable protein diets. Although the Hybrid products were found to be acceptable, this does not mean that the consumers have any intention to buy and further studies should be conducted to determine this type of consumer behavior.

## 5 CONCLUSIONS

Consumer testing has shown that the new concept products are generally well accepted by predominantly meat eaters. Acceptability scores are able to show that the Hybrid concept helps to bridge the gap between meat and meat‐free products. No significant difference in acceptability could be seen between meat samples and Hybrid samples (*p* > .05). This can provide encouragement for the use of the Hybrid concept to reduce consumers’ meat consumption and promote the substitution of meat in consumers’ diets to more sustainable protein sources.

Hybrid sausages were found to have a larger impact on acceptability compared to burgers. Information on this difference is provided by the CATA questions as the acceptability of the burgers was reduced by the samples being too dry as fat and moisture were easily cooked out while in the sausages fat and moisture were retained within the skins. In future reformulations, this issue should be addressed to optimize acceptability.
